# Hospital Meetings, &c.

**Published:** 1897-04-10

**Authors:** 


### HOSPITAL MEETINGS, &C.

### NORTH LONDON HOSPITAL FOR CONSUMPTION.

The annual Court of Governors was held at the board-room, 41, Fitzroy Square, W., on Wednesday, 31st ult., Mr. F. D. Mocatta in the chair. In moving its adoption, the Chairman reviewed the principal items in the report. Amongst others, he recorded the increase in annual subscriptions from £1,616 to £1,787—a gratifying statement, which, however, is somewhat neutralised by the falling off in some of the other sources of income, and the indebtedness to the bankers of £500, to tradesmen of £1,390, and mortgages on the buildings to the tune of £10,500. 441 in-patients and 3,420 out-patients (1,010 of whom attended at Hampstead and the remainder at Fitzroy Square) were treated during the year. The total expenditure has been reduced, and the report gives the following figures as the cost per occupied bed during the past three years: 1894, £106 3s. 6d.; 1895, £96 10s. 6d.; and 1896, £86 18s. Room could be made in the hospital as it now exists for 20 more beds for in-patients, but the difficulty heretofore experienced in raising sufficient funds to maintain those now open has prevented the committee providing them. Mr. Fletcher seconded, and the report was adopted. Lord Kinnaird was elected vice-president, the auditors and the retiring members of the committee were re-elected, and the election as members of the committee of Mr. John Balfour and Colonel G. Grant Gordon, C.B., was confirmed. A vote of thanks to the chairman for presiding completed the business of the meeting.

### NATIONAL HOSPITAL FOR THE PARALYSED AND EPILEPTIC.

This hospital, to which patients from nearly 1,700 cities, towns, and villages of the United Kingdom and its dependencies have been admitted, held its biennial festival at the Whitehall Rooms on Friday, the 2nd inst., H.R.H. the Duke of Connaught in the chair. The special amounts for which an appeal was made were £6,000 for the rearrangement and enlargement of the department for out-patients, which was built 15 years ago; £2,000 for the acquisition of an open space to be used as an airing court for male patients; £1,000 to improve the accommodation for nurses; and £1,000 for the completion of the building fund of the new country and convalescent branch at East Finchley. The Chairman, in proposing the toast of the evening, said it was not so many years ago since his beloved brother stood in his place as president at the festival dinner. He then addressed those present in most eloquent words on behalf of the hospital, but, alas! he was not there to see the good result that followed, and he (the speaker) thought he might congratulate not only the hospital, but the public and the patients on the results of his brother's presidency. During the 35 years of its existence the hospital had advanced in utility. He (the speaker) hoped that in the future the hospital would flourish as it had done in the past, and that its efforts might be crowned with increased success. The hospital had so far never been in debt, but now it required the large sum of £10,000, and he hoped that, as he had never before appealed in vain, the result of this dinner would be the subscription of the sum wanted. Among the other speakers were: Vice-Admiral Lord Charles Scott, the Rev. Canon Duckworth, the Right Hon. Evelyn Ashley, P.C., Sir W. H. Broadbent, Sir J. Crichton Browne, and Cardinal Vaughan. Subscriptions amounting to £3,016 were announced, the chairman's list totalling £2,865.

### ROYAL ORTHOPÆDIC HOSPITAL.

The report presented to the annual meeting of the Royal Orthopædic Hospital, held in the Board Room, on Wednesday, 31st ult., Sir Walter Gilbey, Bart., in the chair, states that owing to a further falling-off in 1896 in the receipts, and an unavoidable increase in the expenditure, the deficit for the year was nearly double that of the previous year, being £396 against £202. Subscriptions and donations had decreased from £976 to £729. 153 in-patients and 706 out-patients were admitted to treatment during the year, the figures for 1895 being 134 and 704 respectively. The average stay of a patient was 104 days. The question of rebuilding the hospital has been under consideration, and a scheme for this purpose has been prepared.

### BRITISH AND FOREIGN SAILORS' SOCIETY.

A meeting in support of the home and foreign work of the British and Foreign Sailors' Society was held on Tuesday, 6th inst., by permission of the Hon. W. F. D. and Lady Esther Smith, at 3, Grosvenor Place, S.W. The Bishop of Stepney, who took the chair in the absence of the Hon. W. F. D. Smith, M.P., said that if there was any class of men to whom Englishmen owed more than they did to any other, it was to sailors, and therefore an association like the British and Foreign Sailors' Society, the first in this or any other country to establish missions and other kindred institutions for the religious and social benefit of sailors, should appeal with great force to them. The Archdeacon of London said that the primary idea of the society was to provide mission-halls, institutes, and rests for sailors at the more important ports. The society also provided floating libraries in all docks and for all seas. The organising secretary (the Rev. C. Parkhurst Baxter) said it was computed that the Thames alone had a floating population of 300,000 sailors and bargemen. A new sailors' rest and reading-room was greatly needed at the Surrey Commercial Docks in South London, and for funds to provide this and for carrying on the general work of the society (which had over 100 stations in the Mediterranean, the North Sea, South America, Australia, India, China, and other distant sea ports) that an appeal was now being made.

### CLAPHAM MATERNITY HOSPITAL.

The eighth annual meeting of the Clapham Maternity Hospital was held on Tuesday last, March 30th, in the board-room of the hospital. Lady Henry Somerset, who had been expected to preside, was, at the last moment, unable to attend. The chair was therefore taken by Mrs. Pearsall-Smith, who, in her opening address, expressed her warm interest in, and sympathy with, the work of the hospital and her approval of the system by which such work is placed entirely in the hands of women. Dr. Helen Webb was re-elected as chairman, and three new members elected on the committee in place of those who had resigned. The general report for 1896 showed an increase both in work and in expenditure. 1,168 maternity cases had been attended during the year, 355 in the hospital and 813 in their own homes in Clapham or Battersea, each patient having received not less than 10 visits. The audience was reminded that the institution also included three dispensaries, some distance apart from each other, where women could see doctors of their own sex, for themselves, or their children, free of charge. During the year 1896 there had been 5,025 such patients (15,666 attendances). The accounts, which are kept on the system approved by the Hospital Sunday and Saturday Funds, and audited each year by chartered accountants, show an expenditure for all these branches of work, of £2,230, of which only £123 was spent in management, the remainder being entirely the cost of maintenance; while the receipts amounted to £2,111, more than two-thirds of which were the proceeds of the Nursing School and patients' payments. On the ground that the hospital is so largely self-supporting, the hon. treasurer earnestly appealed not only for aid in paying off the deficit of £116, with which the year had closed, but for increased help in providing the one-third of the funds for which the hospital is each year dependent upon the subscriptions and donations of any who are willing to "help those who help themselves." The Hon. Mrs. Talbot, wife of the Bishop of Rochester, and Miss Rodbur-Horton, guardian of the poor for the parish of Lambeth, spoke in warm terms of the good work done by the institution. A plea was brought forward not only for further monetary support, but also for gifts in kind, all baby-clothes, old linen and sheeting, and bottles for the dispensaries being most gratefully received. Mrs. Pearsall-Smith brought the meeting to a close by requesting all present to do their utmost to spread abroad a knowledge of the much-needed work which the hospital is doing, and by wishing it long-continued success.

